# Understanding child disability: Factors associated with child disability at the Iganga-Mayuge Health and Demographic Surveillance Site in Uganda

**DOI:** 10.1371/journal.pone.0267182

**Published:** 2022-04-15

**Authors:** Nukhba Zia, Abdulgafoor M. Bachani, Dan Kajungu, Edward Galiwango, Mitchell Loeb, Marie Diener-West, Stephen Wegener, George Pariyo, Adnan A. Hyder

**Affiliations:** 1 Johns Hopkins International Injury Research Unit, Health Systems Program, Department of International Health, Johns Hopkins University Bloomberg School of Public Health, Baltimore, Maryland, United States of America; 2 Iganga-Mayuge Health and Demographic Surveillance Site, Makerere University School of Public Health, Kampala, Uganda; 3 Washington Group on Disability Statistics, Hyattsville, Maryland, United States of America; 4 Department of Biostatistics, Johns Hopkins Bloomberg School of Public Health, Baltimore, Maryland, United States of America; 5 Department of Physical Medicine and Rehabilitation, Division of Rehabilitation Psychology and Neuropsychology, Johns Hopkins School of Medicine, Baltimore, Maryland, United States of America; 6 Department of International Health, Johns Hopkins Bloomberg School of Public Health, Baltimore, Maryland, United States of America; 7 Milken Institute School of Public Health, George Washington University, Washington, DC, United States of America; University of Southern Queensland, AUSTRALIA

## Abstract

**Introduction:**

There is scarcity of data on children with disabilities living in low-and-middle-income countries, including Uganda. This study describes disability prevalence and explores factors associated with different disability categories. It highlights the value of using a standardized, easy-to-use tool to determine disability in children and contextualizing disability in children in light of their developmental needs.

**Methods:**

A cross-sectional study was conducted between September 2018–January 2019 at the Iganga-Mayuge Health and Demographic Surveillance Site in Uganda. Respondents were caregivers of children between 5–17 years and were administered an in-depth Child Functioning Module (CFM). The outcome variable, disability, was defined as an ordered categorical variable with three categories–mild, moderate, and severe. Generalized ordered logit model was applied to explore factors associated with disability categories.

**Results:**

Out of 1,842 caregivers approached for the study, 1,439 (response: 78.1%) agreed to participate in the study. Out of these 1,439, some level of disability was reported by 67.89% (n = 977) of caregivers. Of these 977 children with disability, 48.01% (n = 692) had mild disability and 15.84% (n = 228) had moderate disability, while 3.96% (n = 57) had severe disability. The mean (SD) score for mild disability was 2.22±1.17, with a median of 2. The mean and median for moderate disability was 5.26±3.28 and 4 (IQR:3–6), and for severe disability was 14.23±9.51 and 12 (IQR:6–22). The most common disabilities reported were depression (54.83%) and anxiety (50.87%). Statistically significant association was found for completion of immunization status and school enrollment when controlled for a child’s age, sex, having a primary caregiver, age of mother at child’s birth, family system, family size and household wealth quintile.

**Conclusion:**

This study suggests association between incomplete immunization status and school enrollment for children with disability. These are areas for further exploration to ensure inclusive health and inclusive education of children with disabilities in Uganda.

## Introduction

The World Health Organization (WHO) and United Nations Children’s Fund (UNICEF) regard disability as a human rights issue. Across the globe, disability is considered a major development priority, especially in low-and-middle-income countries (LMICs) [1, 2]. Five out of 17 Sustainable Development Goals (SDGs) specifically focus on disability: Goal 4 focuses on inclusive education; Goal 8 on equal and inclusive employment opportunities; Goal 10 on social, economic and political inclusion of individuals with disabilities; Goal 11 on accessible cities, transport services and public spaces; and Goal 17 emphasizes the importance of high quality, reliable and timely availability of disability data to monitor the progress of other disability-related SDGs [3, 4].

Disability is a complex phenomenon. Based on the International Classification of Functioning, Disability and Health (ICF), disability stems from dynamic interactions between impairments at the body level, within the context of an individual’s health condition and environment, and limitation in an individual’s ability to participate and perform activities [5]. However, measurement of disability at the population level has been particularly problematic due to the complexity of the disability phenomenon [6–8]. Furthermore, assessment of disability among children becomes even more complex, as it is complicated by their non-uniform growth and developmental trajectories. As a result, disability among children is not well understood in LMICs from a public health perspective. The WHO modified the ICF framework and adapted it for children and youth (ICF-CY) to better understand the needs of children with disability as they grow [6–8]. The framework was proposed in 2007 to account for disability in children, which differs from adults in terms of their anatomy and body functioning. The needs and requirements of children change as they go through the various stages of development, especially during early years of life.

One of the main problems in measuring disability among children has been the lack of standardized, easy-to-use instruments that could be used to measure child disability [9, 10]. This has led to the lack of comparable prevalence estimates and hampers the development and evaluation of appropriate policies and programs to address the needs of children with disabilities [9]. To address this gap, UNICEF and the Washington Group on Disability Statistics (WG) developed a disability tool for assessing child functioning (hereafter referred to as the Child Functioning Module–CFM) [11]. The CFM is based on the ICF-CY framework and assesses child functioning among children 2–17 years of age. A sub-module for school-aged children 5–17 contains 24 questions across 13 domains: vision, hearing, walking, self-care, communication, learning, remembering, concentration, accepting change, behavior control, making friends, anxiety and depression [12, 13]. A modified version of the UNICEF/WG tool for 5-17-year-old children was validated in school settings in Fiji in 2015, with the objective to determine if the UNICEF/WG tool can be used by teachers to identify children at risk of disability for timely referral for further assessment and interventions [14, 15]. The CFM underwent cognitive testing in India, Belize, Oman, Montenegro, USA and Jamaica; and field testing in Samoa, El Salvador, Mexico and the final version was tested extensively in Serbia. Additional independent testing has taken place in Haiti, Cameroon, India, Italy and Zambia [1, 11, 12, 16].

Estimates from the WHO put the global prevalence of disability at 15%, or one in seven people in the world; globally, there are about 93 million (one in 20) disabled children less than 15 years of age living with moderate or severe disability [1, 2]. About 90% of these children live in LMICs [1, 2, 8, 10, 17]. The estimated prevalence of moderate to severe disability in the African region is 15.3% [10]. The main causes of disability in the African region include infectious diseases such as polio and leprosy; noncommunicable diseases like congenital malformation and cerebral palsy; injuries such as road traffic crashes; and health-services errors such as inappropriate treatment [18]. However, it is important to point out that the World Report on Disability acknowledges that these numbers are an underestimation and that reliable data on disability–prevalence, type, and causes- are lacking for most LMICs [10].

Uganda is a nation of 42.8 million people located in East Africa [19]. The population of the country is young, with a median age of 15.8 years and life expectancy of 65.7 years at birth [20, 21]. The burden of disease in children between 5–19 years of age is still mainly attributed to communicable diseases like HIV, malaria, and diarrhea. However, non-communicable diseases like skin diseases and asthma, injuries from road crashes, drowning and falls are also contributing to the disease burden [20]. This “triple burden” of diseases (communicable, non-communicable and injuries) is crippling for a fragile economy when people must pay for their own healthcare. Furthermore, in countries with disabilities occurring in youth with a lack of public rehabilitation facilities, the burden of care falls on families.

The 2002 Uganda Population and Housing Census estimated that there are about 2% disabled children in Uganda, and according to the 2014 census, 12.5% of individuals in Uganda have at least one type of disability [22]. Estimates from the UNICEF and the Ministry of Gender of Uganda put this number at 2.5 million disabled children (13% of the population across all age groups) in the year 2014 [22–24]. Specific to the disabled population in Uganda, as per the 2002 census, 30% of the disabled population comprised of children between 0–17 years of age, and about 42% of these had a physical disability [25]. Previously, researchers in Uganda developed and applied instruments to assess disability among adults at the Iganga-Mayuge Health and Demographic Surveillance Site (IM-HDSS) [26, 27]. To overcome socio-cultural issues impeding the identification of individuals with disability, the WG approach was used to identify individuals with specific limitations in key areas of functioning such as vision, hearing, upper and lower limb mobility, self-care, and communication [26–28]. Through this approach, the prevalence of disability in adults was found to be 9.4% in IM-HDSS, with difficulty in vision being the most common type of disability. Being male, older age, and lower socioeconomic status (SES) were associated with physical disability [26]. Disabled adults were found to have greater difficulty in getting around, completing life activities, and participating in society [27]. One interesting finding from this study was that many of these limitations had been life-long, implying that they began in childhood; this study will expand such measurement efforts to children and will generate data to address the gap that exists in disability studies in LMICs.

The overall goal of this paper is to estimate childhood disability prevalence by disability category and assess factors associated with disability in children living at IM-HDSS. More specifically, the study describes overall and domain-specific functioning assessed using the Child Functioning Module (CFM) and examines the prevalence and extent of disability in this child population by various child, caregiver and household characteristics. The hope is to understand the scope of disability among children living at IM-HDSS and examine child and household-level factors associated with disability among children.

## Materials and methods

### Study site

IM-HDSS is in Eastern Uganda and covers the districts of Iganga and Mayuge managed by the Makerere University Centre for health and population research (MUCHAP). IM-HDSS was established in 2005 as a platform field research and research training within Makerere University. It is a member of the International Network for the Demographic Evaluation of Populations and Their Health (INDEPTH) [29–31]. IM-HDSS follows over 95,000 individuals living in about 18,000 households. It conducts census-level data collection twice a year on key demographic events of births, deaths, pregnancies and their outcomes, and in-and out-migrations [31]. In addition, the site periodically collects data on access to health services, causes of death, relevant socioeconomic and education data, non-communicable diseases and injuries [26]. Since 2005, 21 rounds of data collection have been completed (as of June 2019) [29].

This study was nested within an ongoing study to pilot electronic data collection for injuries and disability in IM-HDSS. The main aim of the parent study was to strengthen local capacity to employ cutting-edge information and communication technology (ICT) for research and training on trauma, injuries, and disability by piloting electronic versions of injury and disability data modules. These modules were implemented between 2008–2009 using paper format during previous studies conducted at IM-HDSS and later integrated into IM-HDSS routine data collection rounds. So far injury and disability modules have been implemented in three IM-HDSS data update rounds [26, 27]. The IM-HDSS relies predominantly on paper-based data collection, [32, 33] and the process from data collection to entry into a database and analysis involves multiple steps [32–34]. However, at the time of data collection, the site was transitioning to electronic data collection for efficient and timely availability of data for analysis. A pilot using tablet-based data collection was conducted in April—June 2017 (round 19), and this provided the sampling frame for the current study on child disability.

### Study tool

Two tools were implemented as part of this study. Their details are provided below:

#### Modified Washington Group short set (mWG-SS)

This study used a modified version of the modified Washington Group short set (mWG-SS) [26, 35] It has 6 questions intended for a brief disability assessment of individuals 5 years and older. Each question uses a 4-level Likert scale (0 = no difficulty, 1 = some difficulty, 2 = a lot of difficulty and 3 = cannot do at all), with scores ranging from 0–18, such that the higher the score, the greater the amount of difficulty. It focuses on activity limitations to identify individuals with disability and covers six domains: vision, hearing, walking, upper body mobility, self-care and communication. Previous studies conducted at IM-DSS and elsewhere found that it takes approximately 10 minutes to administer, and the questions are well understood by respondents [26, 28, 35, 36] The main purpose of this tool is to identify individuals who, because of difficulties doing these basic, universal activities are at greater potential risk than the general population of limitation in social participation their basic life activities (e.g., accessing education or employment, walking, hearing, and vision). The WG previously administered mWG-SS during field testing of CFM in Serbia [37].

It is important to note that mWG-SS had already been translated into the local language, Lusoga, and was implemented at IM-HDSS for disability assessment in individuals 5 years and above at the household level [26, 27] mWG-SS was first introduced at the IM-HDSS in 2009, and2009 and has since then it has been implemented thrice in three more times (2011, 2014 and 2017). Currently, only adults (18 years and older) identified to have disability based on mWG-SS are followed-up using a more detailed disability assessment tool to further characterize the implications of their activity limitation on different life domains. The WHO Disability Assessment Schedule 2.0 (WHODAS 2.0) is used for this purpose and was first implemented at the IM-HDSS in 2011, with another round conducted and later in 2017 [26, 27].

The data collection in round 19 (April–July 2017) was the first time IM-HDSS piloted electronic data collection of mWG-SS and WHODAS 2.0. Since the WHODAS is only applied to individuals over 18 years, disability among children has not been previously studied at the IM-HDSS. The current study focuses on children between 5–17 years of age and is therefore an extension of the current disability work being done at IM-HDSS to allow for a better understanding of disability in this younger age group. Data from mWG-SS was used for validating CFM [38].

#### Child Functioning Module (CFM)

This study utilizes a detailed Child Functioning Module (CFM) developed by UNICEF in collaboration with the Washington Group on Disability Statistics [12]. CFM focuses on basic, everyday activities that relate specifically to child functioning. It can be administered at the national level at part of national household surveys and allows for comparisons across time and countries [12]. It comprises 24 questions, with responses on a 4-level Likert scale (0 = no difficulty, 1 = some difficulty, 2 = a lot of difficulty and 3 = cannot do at all). These questions result in 13 domains, with scores ranging from 0–39; the higher the score, the greater the amount of difficulty. It takes about 20–25 minutes to complete the interview [13]. The 13 CFM domains include vision, hearing, walking, self-care, communication, learning, remembering, concentration, accepting change, behavior, making friends, feeling anxiety, and feeling depression [13].

Additional data collected during this study included information on household head and members, household wealth quintile, child and caregiver demographics (age, gender, education), caregiver education and employment status, childbirth, vaccination, sibling information, school and work history, and health seeking practices. The CFM and additional questions were translated to Lusoga and back translated to English.

### Study design and respondents

This was a *cross-sectional study* conducted between September 2018 –January 2019. Respondents were caregivers of children between 5 to 17 years of age. At the time of the study, there were 35,062 children between the ages of 5–17 years residing in the IM-HDSS. This study focuses ion children between 5–17 years of age since this is the recommended age range for the CFM version that was used for this study.

### Sampling frame and sample size

Sampling frame for this study was drawn from household and individual listings available from the latest IM-HDSS rounds (round 19 and 20). Data from a pilot conducted as part of round 19 served as basis for identifying children with disabilities who were between 5–17 years of age. This was done using data from mWG-SS that was administered at the household level. A total of 377 children between the ages of 5–17 years were identified to have some form of disability based on round 19 mWG-SS data. Their IDs were then confirmed for active status in round 20, which had been completed four months (May 2018) before the beginning of this study (September 2018). Based on the round 20 check for active IDs, 342 children out of 377 from round 19 were found to have active IDs (29 children were more than 17 years, one had died, four had moved to another location within IM-HDSS, and one had moved out of IM-HDSS). Active IDs mean that these children were present at the IM-HDSS as of round 20; hence, all 342 children were included in this study.

In addition to children with disability, a sample size of 1,273 was computed assuming the ability to observe a difference in disability prevalence of 1% between two groups. Other parameters included alpha of 5% and power of 80%. Thus, the total sample required was 1,615 (1,273 children without disability and 342 with disability). However, to account for non-availability, refusals, and out-migrations from the site, sample for non-disabled was increased to 1500. At the time of this study, 35,062 children (excluding 342 with disability) were residing at the IM-HDSS. A stratified (based on sex of child) sample proportionate to population size of children without disability was drawn from the list of 35,062 children. The formula used for the stratified sample size calculation was:

$$\begin{array}{ll} \left(\frac{\text{Total}\;\text{sample}\;\text{size}\;\text{calculated}}{\text{Population}\;\text{size}}\right) & \text{x}\;\text{Stratum}\;\text{size} \end{array}$$

Table 1 below gives sample size calculation for each stratum.

**Table 1:** Sample size calculation.

Sample size calculation	Male	Female
Total sample size	1,500	1,500
Population size	35,062	35,062
Stratum size	17,216	17,846
Calculation	(1,500/35,062) * 17,216	(1,500/35,062) * 1,7846
Stratified sample	737	763

Using simple random sampling, a list of IDs was drawn from each stratum using STATA version 14 [39]. Thus, the sample for this study included 342 children with disabilities and 1,500 children without disabilities, giving a total of 1,842 children whose caregivers were approached to participate in the study. Only one child per household was selected. A unique study ID was assigned to all 1,842 children included in the sample.

**Fig 1:**
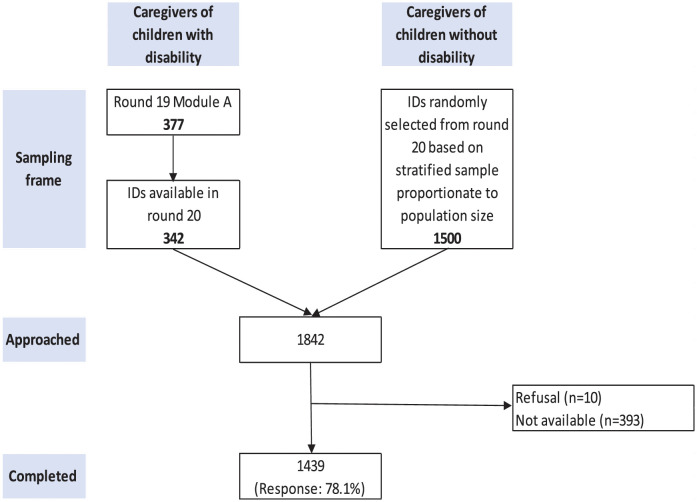
Sampling frame for child disability assessment at the Iganga-Mayuge Health and Demographic Surveillance Site.

It is important to note that the distinction between children with disability and without disability was made for sampling purpose to ensure that sample for this study does not miss children with disabilities. The analysis for this study was conducted on the pooled sample of individuals who agreed to participate in the study. Fig 1 depicts enrollment of caregivers.

### Data collection and management

After obtaining oral informed consent, data were collected through face-to-face caregiver interviews using a tablet-based platform that was developed and pre-tested based on available local technical resources at the IM-HDSS. The platform included English and Lusoga versions of questions, which were developed in KoBoToolbox (https://www.kobotoolbox.org/) for data collection. Questions had check box and free text entry formats to input responses. Questions were designed to allow skip patterns where appropriate, and mandatory fields were also marked. This ensured that there were no missing data for mWG-SS and CFM. In order to mitigate transcription error as a result of re-entry of IM-HDSS IDs, unique study IDs were linked to the individual IM-HDSS IDs. The Kobocollect App was downloaded to android tablets to allow for data collection using user-specific passwords. The forms were accessible in the field during data collection and did not require internet or Wi-Fi connection to complete them. Once an interview was completed, the form was saved on a tablet offline. Field supervisors checked the saved forms at the end of the day before uploading the completed forms to a Cloud sever using HDSS office Wi-Fi connection. The cloud server is secure and encrypted and was only accessible to authorized study team members at IM-HDSS and Johns Hopkins School of Public Health. This ensured data confidentiality and security. Data were downloaded for analysis from the server in MS Excel (.xls and.cvs format).

### Data analysis

#### Outcome variable

The outcome variable of interest was disability and was categorized into four *severity* groups of no disability, mild disability, moderate disability, and severe disability, using 4-point Likert responses of CFM. “No disability” was defined when “no difficulty” was marked on all CFM questions. “Mild disability” was defined when “some difficulty” was marked as the highest response for any one of the CFM domains. “Moderate disability” was defined when “a lot of difficulty” was marked as the highest response for any one of the CFM domains. “Severe disability” was defined when “cannot do at all” was marked as the highest response for any one of the CFM domains. The total CFM score ranged between 0–39. Scores for mild disabilities ranged between 1–13, moderate disability ranged between 2–26, and severe disability ranged between 3–39. This classification was used for further analysis including regression analysis. Table 2 gives definition of disability categories.

**Table 2:** Definition of disability categories.

Disability categories	Definition
**None**	“no difficulty” marked on all domains
**Mild**	“some difficulty” was the highest response for any one domain
**Moderate**	“a lot of difficulty” was the highest response for any one domain
**Severe**	“cannot do at all” was the highest response for any one domain

#### Descriptive analysis

Descriptive analysis was conducted to calculate the prevalence of disability based on disability categories. Child, parent, primary caregiver and household characteristics were explored by level of disability categories. Binary and categorical variables were reported in percentages and mean with standard deviation as well as median and interquartile range (IQR) are reported for continuous variables. Chi square tests were conducted to assess relationships between categorical variables [40]. To assess the relationship between non-normally distributed continuous variables and the ordered categorical disability variable, Kruskal Wallis tests were conducted. This is a non-parametric test comparable to ANOVA, which compares medians of two or more groups [41].

#### Regression analysis

For regression analysis, disability outcome variable with three categories–mild, moderate and severe was considered. Since the outcome variable “disability” was categorical and ordered, the first choice of regression analysis was ordinal logistic regression. This model is based on the proportional odds or parallel-regression assumption, which means that the value of odd ratios in the model remains the same across various categories of the dependent variable [42, 43]. For example, the odd ratio between mild to moderate is the same as between moderate to severe. The proportional odds assumption was tested using two methods. The first is a *likelihood ratio test*, which tests the null hypothesis that there is no difference in the coefficients between models [44]. The second test is the *Brant test of parallel regression assumption*, which checks the proportional odds assumption [45]. The null hypothesis for each of these tests is that of proportionality; rejecting the null hypothesis provides evidence against using the ordinal logistic regression [44]. When the results of these tests are statistically significant (p-value <0.05), the proportional odds assumption is violated, and an alternate model called generalized ordered logit model may be used.

Generalized ordered logit model relaxes the proportional odds assumption and allows estimation of a partial proportional odds model [42, 43]. Furthermore, it presents results by dichotomizing the categorical outcome variable. For generalized ordered logit regression analysis with three categories, there are two panels of odd ratios. The first one compares mild disability with combined moderate and severe, and the second panel compares combined mild and moderate disability with severe disability. This approach allows the relationship with independent variable to vary based on different dichotomization of the categorical outcome variable [42, 43]. The mathematical equation for the generalized ordered logit model is as follows:

$$\begin{array}{ll} P\left(Y_{i}>j\right)= & \frac{\text{exp}\left(\alpha_{\text{j}}+{\text{X}}_{\text{i}}\beta_{\text{j}}\right)}{1+\left[\text{exp}\left(\alpha_{\text{j}}+{\text{X}}_{\text{i}}\beta_{\text{j}}\right)\right]}\\ \text{J}=1,2,\ldots .,\text{M}-1 & \end{array}$$

Independent variables considered in the regression models included the child’s age, sex, immunization status, school enrollment, primary caregiver status, age of mother at child’s birth, type of family system, family size and household wealth quintile. All analysis was conducted in STATA 14 [39].

### Ethical approval

The study was approved by the institutional ethics committees of the Johns Hopkins Bloomberg School of Public Health, USA and both Makerere University School of Public Health and the Uganda National Council for Science and Technology.

## Results

### Descriptive analysis

#### Disability prevalence and scores

Out of 1,842 caregivers approached for the study, 1,439 caregivers (response: 78.1%) agreed to participate in the study (Fig 1). There were 10 refusals (0.69%), while 393 caregivers were not available (27.31%) for interview. Out of these 1,439, some level of disability was reported by 67.89% (n = 977) caregivers. Of these 977 children with a disability, 48.01% (n = 692) had mild disability, 15.84% (n = 228) had moderate disability, and 3.96% (n = 57) had severe disability (Table 3). The CFM mean score for mild disability was 2.22 ± 1.17 out of 13, with a median of 2. The mean and median for moderate disability was 5.26 ± 3.28 out of 26 and 4 (IQR:3–6), and for severe disability, they were 14.23 ± 9.51 out of 39 and 12 (IQR:6–22). (Table 3).

**Table 3:** Mean, median, minimum and maximum CFM scores based on disability categories (n = 1,439).

Disability categories	n (%)	Mean ± SD	Median (IQR)	Minimum	Maximum
**Overall**	1,439	2.47 ± 3.82	2 (0–3)	0	35
**None**	462 (32.11)	0	0	0	0
**Mild**	692 (48.09)	2.22 ± 1.17	2 (0)	1	10
**Moderate**	228 (15.84)	5.26 ± 3.28	4 (3–6)	2	19
**Severe**	57 (3.96)	14.23 ± 9.51	12 (6–22)	3	35

**Fig 2:**
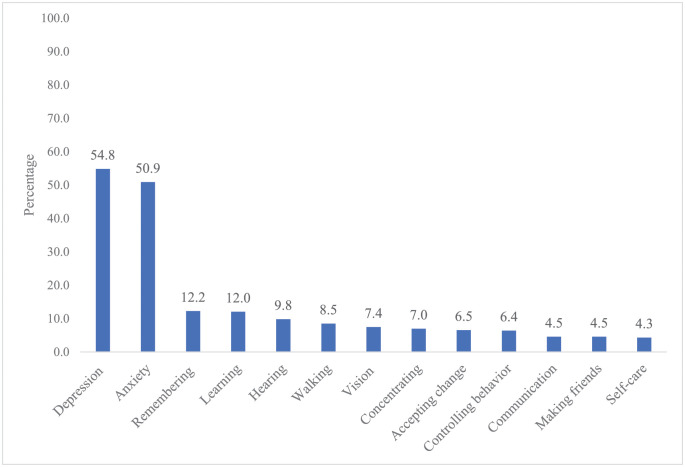
Percentage distribution of overall disability by CFM domains (n = 1,439).

**Fig 3:**
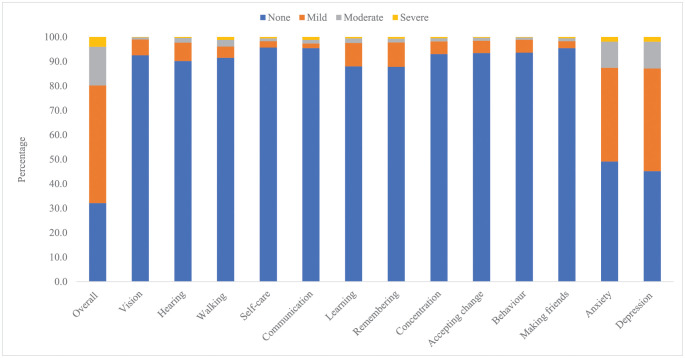
Percentage distribution of disability category by CFM domains (n = 1,439).

Overall, the most common disability was depression (54.83%), followed by anxiety (50.87%) and remembering (12.2%). The least reported disability domains were communication (4.52%), making friends (4.52%), and self-care (4.31%). (Fig 2). Based on the disability categories, more than 90% of the children did not have any disability related to vision, hearing, walking, communication, concentration, accepting change, behavior, and making friends. No disability in learning and remembering was reported for 88% and 87.8% of the children respectively (Fig 3). However, the reported disability related to anxiety or depression was much higher than that of other domains. Overall, 38.3% had mild disability related to anxiety, while 42.0% had mild disability related to depression. Similarly, 10.7% had moderate disability related to anxiety, while 10.9% had moderate disability related to depression.

#### Demographic characteristics

**Fig 4:**
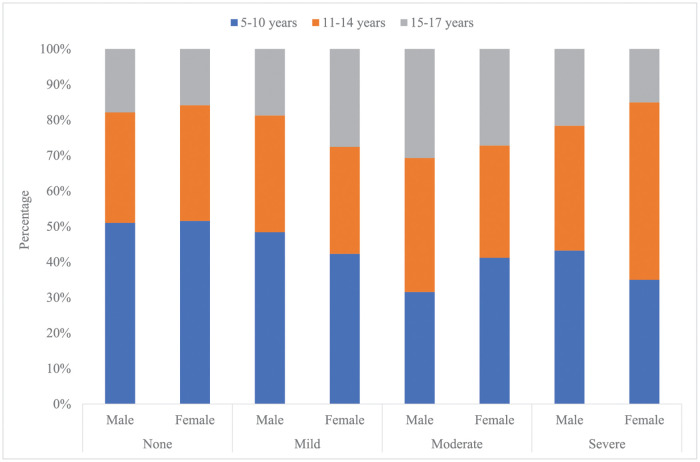
Percentage distribution of age category by disability category and sex of child (n = 1,439).

Most of the children with mild disability were between 5–10 years of age (n = 314, 45.38%), while the distributions of children in the moderate and severe disability groups were almost similar for the 5-10- and 11-14-year age groups. The distribution of children based on child sex varied across the disability groups, with more males (64.91%) in the severe disability group. (Fig 4). Being severely disabled was associated with the lowest percentage of completed immunization (29.41%) (p-value <0.001) and not being enrolled in school (47.37%, p-value <0.001) (Table 4). The respondents identified a primary caregiver for children, with the highest percentage of primary caregivers for children with severe disability (94.74%) compared to those with mild (88.87%) and moderate (94.30%) disability (p-value <0.001) (Table 4).

**Table 4:** Distribution of child, parent, primary caregivers, and household characteristics by disability categories (n = 1,439).

Characteristics	None (n = 462)	Mild (n = 692)	Moderate (n = 228)	Severe (n = 57)	Total (n = 1,439)
**Child Characteristics**					
**Average age in years (mean ± SD)** *	10.52 ± 3.49	11.12 ± 3.71	11.83 ± 3.40	11.47 ± 3.03	11.06 ± 3.59
**Age groups** *					
5–10 years	237 (51.30)	314 (45.38)	83 (36.40)	23 (40.35)	657 (45.66)
11–14 years	147 (31.82)	218 (31.50)	79 (34.65)	23 (40.35)	467 (32.45)
15–17 years	78 (16.88)	160 (23.12)	66 (28.95)	11 (19.30)	315 (21.89)
**Sex**					
Male	241 (52.16)	347 (50.14)	114 (50.00)	37 (64.91)	739 (51.36)
Female	221 (47.84)	345 (49.86)	114 (50.00)	20 (35.09)	700 (48.64)
**Immunization (completed)** *	211 (49.18)	223 (34.31)	108 (49.77)	15 (29.41)	557 (41.35)
**Currently in school (yes)** *	425 (91.99)	624 (90.17)	196 (85.96)	27 (47.37)	1,272 (88.39)
**Siblings (yes)**	442 (95.67)	670 (96.82)	221 (96.93)	55 (96.49)	1,388 (96.46)
**Have a primary caregiver (yes)** *	355 (76.84)	615 (88.87)	215 (94.30)	54 (94.74)	1,239 (86.10)
**Parental Characteristics**					
**Mother alive (yes)**	446 (96.54)	674 (97.40)	218 (95.61)	53 (92.98)	1,391 (96.66)
**Mother’s age at child’s birth in years (mean ± SD)**	25.72 ± 6.37	25.28 ± 6.40	25.49 ± 7.00	26.54 ± 7.46	25.50 ± 6.53
**Mother’s education level** *					
None	58 (12.55)	48 (6.94)	10 (4.39)	10 (17.54)	126 (8.76)
Primary	263 (56.93)	396 (57.23)	149 (65.35)	37 (64.91)	845 (58.72)
Lower secondary	89 (19.26)	176 (25.43)	49 (21.49)	4 (7.02)	318 (22.10)
Upper secondary	4 (0.87)	3 (0.43)	1 (0.44)	-	8 (0.56)
Other (university/vocational)	48 (10.39)	69 (9.97)	19 (8.33)	6 (10.53)	142 (9.87)
**Mother’s occupation** *					
Housewife	52 (11.26)	104 (15.03)	22 (9.65)	12 (21.05)	190 (13.200
Farmer	241 (52.16)	321 (46.39)	125 (54.82)	32 (56.14)	719 (49.97)
Shopkeeper	100 (21.65)	167 (24.13)	60 (26.32)	8 (14.04)	335 (23.28)
Professional	17 (3.68)	29 (4.19)	8 (3.51)	2 (3.51)	56 (3.89)
Unemployed	8 (1.73)	22 (3.18)	5 (2.19)	-	35 (2.43)
Others	28 (6.06)	24 (3.47)	5 (2.19)	1 (1.75)	58 (4.03)
**Father alive (yes)**	431 (93.29)	649 (93.79)	207 (90.79)	54 (94.74)	1,341 (93.19)
**Father’s education level** *					
None	32 (6.93)	15 (2.17)	8 (3.51)	5 (8.77)	60 (4.17)
Primary	196 (42.42)	308 (44.51)	108 (47.37)	37 (64.91)	649 (45.10)
Lower secondary	105 (22.73)	236 (34.10)	71 (31.14)	4 (7.02)	416 (28.91)
Upper secondary	12 (2.60)	21 (3.03)	8 (3.51)	1 (1.75)	42 (2.92)
Other (university/vocational)	117 (25.32)	112 (16.18)	33 (14.47)	10 (17.54)	272 (18.90)
**Father’s occupation** *					
Farmer	153 (33.12)	186 (26.88)	86 (37.72)	19 (33.33)	444 (30.85)
Shopkeeper	131 (28.35)	248 (35.84)	68 (29.82)	17 (29.82)	464 (32.24)
Professional	44 (9.52)	37 (5.35)	16 (7.02)	3 (5.26)	100 (6.95)
Boda boda driver	41 (8.87)	87 (12.57)	25 (10.96)	7 (12.28)	160 (11.12)
Unemployed	5 (1.08)	2 (0.29)	2 (0.88)	-	9 (0.63)
Others	72 (15.58)	116 (16.76)	26 (11.40)	10 (17.54)	224 (15.57)
**Primary caregiver characteristics**					
**Caregiver relation with child** *					
Mother	196 (55.21)	407 (66.18)	133 (61.86)	34 (62.96)	770 (62.15)
Father	64 (18.03)	71 (11.54)	20 (9.30)	9 (16.67)	164 (13.24)
Grandparent(s)	61 (17.18)	76 (12.36)	31 (14.42)	5 (9.26)	173 (13.96)
Uncle/Aunt	10 (2.82)	31 (5.04)	14 (6.51)	3 (5.56)	58 (4.68)
Sibling	7 (1.97)	4 (0.65)	2 (0.93)	-	13 (1.05)
Others	16 (4.51)	26 (4.23)	15 (6.98)	3 (5.56)	61 (4.92)
**Average age (mean ± SD)** *	41.91 ± 12.17	40.31 ± 11.60	42.17 ± 11.54	41.56 ± 9.65	41.15 ± 11.69
**Sex**					
Male	68 (19.15)	106 (17.24)	32 (14.88)	13 (24.07)	219 (17.68)
Female	287 (80.85)	509 (82.76)	183 (85.12)	41 (75.93)	1,020 (82.32)
**Education level** *					
None	71 (20.00)	70 (11.38)	17 (7.91)	10 (18.52)	168 (13.56)
Primary	196 (55.21)	353 (57.40)	143 (66.51)	39 (72.22)	731 (59.00)
Lower secondary	60 (16.90)	144 (23.41)	43 (20.00)	3 (5.56)	250 (20.18)
Upper secondary	3 (0.85)	6 (0.98)	3 (1.40)	-	12 (0.97)
Other (university/vocational)	25 (7.04)	42 (6.83)	9 (4.19)	2 (3.70)	78 (6.30)
**Occupation**					
Farmer	200 (56.34)	314 (51.06)	130 (60.47)	35 (64.81)	679 (54.80)
Shopkeeper	78 (21.97)	144 (23.41)	48 (22.33)	11 (20.37)	281 (22.68)
Housewife	25 (7.04)	78 (12.68)	12 (5.58)	5 (9.26)	120 (9.69)
Professional	19 (5.35)	28 (4.55)	7 (3.26)	1 (1.85)	55 (4.44)
Boda boda driver	3 (0.85)	6 (0.98)	3 (1.40)	1 (1.85)	13 (1.05)
Unemployed	11 (3.10)	12 (1.95)	6 (2.79)	1 (1.85)	30 (2.42)
Others	19 (5.35)	33 (5.37)	9 (4.19)	-	61 (4.92)
**Household Characteristics**					
**Family system** *					
Single parent	64 (13.85)	51 (7.37)	16 (7.02)	7 (12.28)	138 (9.59)
Nuclear	242 (52.38)	364 (52.60)	110 (48.25)	27 (47.37)	743 (51.63)
Joint	155 (33.55)	277 (40.03)	101 (44.30)	22 (38.60)	555 (38.57)
**Family size (mean ± SD)**	7.77 ± 2.93	7.88 ± 3.10	8.13 ± 3.65	8.09 ± 3.69	7.89 ± 3.16
**Household wealth quintile**					
Poorest	122 (27.23)	189 (28.51)	63 (28.25)	15 (27.27)	389 (28.01)
Poorer	93 (20.76)	137 (20.66)	58 (26.01)	11 (20.00)	299 (21.53)
Poor	108 (24.11)	151 (22.78)	51 (22.87)	15 (27.27)	325 (23.40)
Less poor	85 (18.97)	121 (18.25)	33 (14.80)	9 (16.36)	248 (17.85)
Least poor	40 (8.93)	65 (9.80)	18 (8.07)	5 (9.09)	128 (9.22)

* p-value <0.05.

The mean age of mothers at the time of birth of their child was around 25 years, which was slightly higher for mothers of children with severe disability (Table 4). The percentage of uneducated parents was higher for children with severe disability (mother: 17.54%, father: 8.77%), while it was much lower for mild (mother: 6.94%, father: 2.17%) and moderate (mother: 4.39%, father: 3.51%) disability groups, although over 50% of mothers and over 40% of fathers had received primary education. About 18% of primary caregivers of children with severe disability were uneducated while almost 70% had received primary education (Table 4).

About 12.28% of children with severe disability lived with single parents, while 7% of children with mild and moderate disability had single parents (Table 4). Children with mild to moderate disability had a higher percentage living in nuclear and joint family systems compared to those with severe disability (p-value <0.001).

#### Regression analysis

This study analyzed factors associated with disability using generalized ordered logit model. The decision was based on statistically significant (p-value <0.05) findings of the Brant test, which indicated that the proportional odds assumption was violated for the ordinal regression model and depicted that the relationships between disability and independent variables in the model vary across the different disability categories.

According to the results of the generalized ordered logit model, completion of immunization status and school enrollment were statistically and significantly associated with disability when controlled for the child’s age, sex, primary caregiver status, age of mother at child’s birth, type of family system, family size and household wealth quintile (Table 5).

**Table 5:** Factors associated with disability in children living at the IM-HDSS, Uganda (Generalized ordered logistic model).

Characteristics	Mild versus combined moderate and severe		Combined mild and moderate versus severe	
	Odd ratios (95% CI)	Coefficient (95% CI)	Odd ratios (95% CI)	Coefficient (95% CI)
**Age groups**				
5–10 years	Reference		Reference	
11–14 years	1.57* (1.11–2.25)	0.46* (0.10–8.1)	1.33 (0.64–2.75)	0.28 (-0.44–1.01)
15–17 years	1.41 (0.94–2.09)	0.34 (-0.06–0.74)	0.49 (0.21–1.14)	-0.72 (-1.58–0.13)
**Sex**				
Male	Reference		Reference	
Female	0.91 (0.67–1.24)	-0.09 (-0.40–0.21)	0.57 (0.30–1.10)	-0.56 (-1.21–0.09)
**Immunization status**				
Completed	Reference		Reference	
Not completed	0.58* (0.42–0.80)	-0.54* (-0.86–-0.22)	2.36* (1.12–4.96)	0.86* (0.12–1.60)
**Currently in school**				
Yes	Reference		Reference	
No	1.60* (1.01–2.53)	0.47* (0.01–0.93)	11.01* (5.15–23.56)	2.40* (1.64–3.16)
**Have a primary caregiver**				
Yes	Reference		Reference	
No	0.40* (0.21–0.79)	-0.91* (-1.58–-0.23)	0.70 (0.15–3.18)	-0.36 (-1.87–1.16)
**Mother’s age at birth**				
14–20 years	Reference		Reference	
21–30 years	0.89 (0.62–1.28)	-0.11 (-0.48–0.25)	0.73 (0.33–1.61)	-0.31 (-1.10–0.48)
31–40 years	1.02 (0.64–1.62)	0.02 (-0.45–0.48)	1.37 (0.59–3.19)	0.32 (-0.53–1.16)
41–50 years	1.22 (0.50–2.95)	0.20 (-0.69–1.08)	0.45 (0.08–2.47)	-0.79 (-2.49–0.90)
**Family system**				
Single parent	Reference		Reference	
Nuclear	1.14 (0.64–2.06)	0.13 (-0.45–0.72)	0.56 (0.19–1.63)	-0.59 (-1.67–0.49)
Joint	1.54 (0.83–2.88)	0.43 (-0.19–1.06)	0.50 (0.16–1.58)	-0.69 (-1.84–0.46)
**Family size**	1.01 (0.97–1.06)	0.01 (-0.04–0.06)	1.08 (0.99–1.18)	0.08 (-0.01–0.17)
**Household wealth quintile**				
Poorest	Reference		Reference	
Poorer	1.24 (0.81–1.88)	0.21 (-0.20–0.63)	0.67 (0.27–1.68)	-0.40 (-1.31–0.52)
Poor	0.97 (0.63–1.48)	- 0.32 (-0.46–0.39)	1.61 (0.67–3.88)	0.48 (-0.40–1.36)
Less poor	0.88 (0.54–1.44)	-0.13 (-0.62–0.37)	1.62 (0.58–4.49)	0.48 (-0.54–1.50)
Least poor	0.86 (0.48–1.53)	-0.15 (-0.73–0.42)	1.04 (0.33–3.27)	0.04 (-1.10–1.18)

*Statistically significant at p-value <0.05

#### Mild disability versus combined moderate and severe disability

After controlling for the independent variables in the model, comparing children in mild disability group to higher disability categories (combined moderate and severe disability) showed that the children with higher disability were 42% less likely to have incomplete immunization (aOR = 0.58; 95% CI: 0.42–0.80). Comparing children in mild disability category to higher disability categories showed that children in higher categories were 1.60 times more likely to be out of school (aOR = 1.60; 95% CI: 1.01–2.53). The adjusted odds of not having a primary caregiver were 60% less for children with mild disability (aOR = 0.40; 95% CI: 0.21–0.79) (Table 5).

#### Mild and moderate disability versus severe disability

After controlling for the independent variables in the model, comparing children in mild and moderate disability groups to those in the severe disability category showed that children with severe disability were 2.36 (95% CI: 1.12–4.95) times more likely to have incomplete immunization. Comparing children in mild and moderate disability groups to the severe disability category showed that children with severe disability were 11.01 times more likely to be out of school (aOR = 11.01; 95% CI: 5.15–23.56).

## Discussion

To the best of our knowledge, this is the first study conducted at IM-HDSS to assess prevalence of disability among children and factors associated with disability among children between 5–17 years of age. Depending on how disability is defined, it was found that between 58.37%–67.89% children living at IM-HDSS have some form of disability. Previous studies done to estimate disability prevalence in different context report different percentages based on disability definition. For example, prevalence was reported to be 63.3% based on the most inclusive definition of disability (“some difficulty” in one of the CFM domains); however, it was reported to be 42.0% when disability was defined as “some difficulty” in two of the CFM domains, 8.9% for moderate and 0.7% for severe [46]. The same study reported that at least 34.5% of the children in India had some form of disability [46]. While another study reported this prevalence to be at 46.3% for Mexico, 9.8% for Samoa, and 25.2% for Serbia [16].

It is important to note that the distribution of disability categories followed a positively skewed distribution, with more children found to have mild disability and a very small number with severe form of disability. This has implications for developing and implementing targeted interventions. According to recommendations by UNICEF/WG, disability is categorized as a binary variable, and several different cut-offs are suggested [16, 37]. However, for this study, disability was categorized based on Likert-responses to allow assessment of factors without losing granularity in the data. This analysis shows that while the definition of moderate and severe disability is clear, defining mild disability is a challenge. Based on the sensitivity analysis, it was found that by changing the definition of mild disability, disability prevalence for this category changed by 10%. This means that disability prevalence in this population might be overestimated for children at IM-HDSS. Thus, caregiver responses that place children into the “some difficulty” category needs careful consideration. This could be due to some behavioral changes that the child is going through instead of actually having disability [37]. This may have increased false positives and, thus, overestimated disability prevalence. But at the same time, it provides an in-depth assessment of children at the community level. This is vital for early identification of disability and for monitoring the progress of these children in case an intervention is implemented.

The factors considered for association with disability included selected child, primary caregiver and household characteristics. Incomplete child immunization status and lack of school enrollment at the time of the study were found to be associated with disability. It is important to note that due to the cross-sectional study design, this study only shows association of disability with immunization status and school enrollment. It does not give casual relation between disability and these variables. Disability can have devastating effects on individuals, their families and the society. It is regarded as a cause and consequence of poverty. In many LMICs, disability is still highly stigmatized and can lead to social exclusion and discrimination. For children with disability, this often means a life in isolation and exclusion from education and future employment opportunities as well as a lack of access to health services [2, 10, 17, 47–49].

An important finding in this study is the association of disability with completion of immunization. It was noticed that children with mild disability seemed to have higher odds of incomplete immunization compared to children in higher disability categories. This is an interesting finding; it may reflect that children with moderate and severe disability have greater access to health services and, thus, are able to receive these immunizations. However, when children with mild to moderate disability were compared to those with severe disability, children with severe disability were less likely to have had completed their immunizations. This opposing trend may be driven by how children with moderate disability are placed and could also be due to the small number of children with severe disability. However, the study highlights the importance of providing preventive measures for vaccine-preventable diseases and the need to address access to immunization [50]. There may be several contributing factors, including lack of vaccines, trained healthcare providers, and ramps for wheelchairs for children with disabilities at the designated immunization centers and facilities; or the burden on the family and caregiver to take the children for vaccination perhaps due to the high cost associated with transportation; or the attitude of providers even if the child is taken for vaccination. Stigmatization and lack of trust in the healthcare system coupled with lack of resources could be a reason for incomplete immunization [50, 51].

School enrollment for children with mild and moderate disability was higher as compared to those with severe disability. In Uganda only 9% of children with mild and moderate disability attended primary school, and 6% attended secondary school [23]. Of the children with disabilities in Uganda, only 10% have access to schools that meet their needs, and only 5% of disabled children going to public school receive specialized education [24]. These estimates are worrisome and underscore the significant impact that disability can have on the development and future life opportunities for these children. Another report from Uganda, however, shows that about 62% of disabled children between 9–17 years of age are enrolled in school [25]. With relatively high enrollment reported at the IM-HDSS, a potential area to focus on in future work is to train teachers to teach children with disabilities and providing accommodations to receive education. This may include having accessible school transportation, ramps within school buildings, accessible toilet facilities, and study aids for children with visual and hearing impairments [52].

In addition to assessing overall disability, all 13 specific domains were also assessed for disability categories. Higher percentages of disability were reported for anxiety and depression compared to other domains. This finding is consistent with Massey et al work in the US [48]. This could be due to lack of understanding of questions by caregivers, or it may depict an underlying feeling of sadness and unhappiness as perceived by caregivers [53]. How caregivers perceive level of anxiety and depression among their children may be a reflection of their own level of anxiety and depression, which might be projected in their responses to the questions related to anxiety and depression [54]. In either case, this is an interesting finding which needs further exploration using in-depth clinical assessments for anxiety and depression to ensure that children receive proper treatment and therapy. This approach can also help to identify at risk population of children who might be influenced by adverse household and social factors like loss of a parent or being marginalized [53].

### Strengths and limitations

Some strengths of this study are it is the first study at IM-HDSS and Uganda that has assessed disability in children and its associated factors. This is a priority area for the local district health office and will help in developing, implementing, and monitoring relevant interventions. Second, CFM assesses physical as well as developmental limitations in children, thus acknowledging growth and developmental needs of children with disability. Third, the tool is validated in Ugandan context and can be administered in a community setting by non-clinical staff to assess the burden of disability in the community [38, 55]. The IM-HDSS staff works very closely with the local district health office and can use data from the child disability module to ensure integration of children into the education and healthcare systems. Fourth, the tool asks for level of difficulty associated with CFM domains but does not label children as “disabled.” This helps to avoid stigmatization of children with disabilities and reduces any level of discomfort a caregiver might have faced during the interview. Fifth, the analysis assesses the relationship between disability utilizing Likert-scale responses and associated factors in bivariate analyses and accounted for ordered responses during the multivariable regression analysis. Sixth, this study does not report specific causes of disability; instead, it focuses on domains which, according to the ICF framework, constitute activities and ability of children to function in their daily life. However, future work should explore specific causes of disability and their association with the disability categories.

This study explored factors associated with disability only for children living at the IM-HDSS; these factors may differ from other regions in Uganda and in other LMICs. It is, therefore, not possible to generalize findings from this study to the Ugandan context, but the results provide an understanding of disability burden at the site as well as an opportunity for data sharing with the local district health office to plan inclusive interventions in the districts. Second, disability categorization into mild, moderate and severe may have overestimated disability prevalence at the IM-HDSS. However, this allows opportunity for early identification and intervention for children with mild disability who may progress to moderate or even severe disability without any intervention. Third, this study enrolled children with and without disability. This might have also contributed to overestimation of disability prevalence at the IM-HDSS, but it also shows that disability is a dynamic process and that children who were previously reported to have disability did not report disability during this study and vice versa. Thus, it is important that longitudinal studies are conducted to understand the dynamic disability process and its impact on growth and development of children. Fourth, the study highlights that children with disability lack opportunities related to education and healthcare access. However, due to the inherent limitations of the cross-sectional study design, it was not possible to explore the causal relationship between immunization and disability, and between school enrollment and disability. Future work needs to focus on exploring limitations related to school and healthcare access.

Fifth, CFM assessed 13 domains comprising physical functioning and developmental aspects. However, there was no clinical assessment done to verify limitations reported by caregivers. It is important that CFM is implemented as a tool in the community to facilitate early identification of children with disabilities so that they can be referred for clinical assessment and intervention in a timely manner. Sixth, since CFM is a self-reported tool, there is a possibility of bias in caregiver responses prompted by perceived gains, for example in terms of enrollment into intervention studies. However, the structure of questions in CFM allows an assessment of the level of difficulty without labeling children as disabled. Future work should allow longitudinal data collection to determine change in disability status of children and possible adaptations as a result of their disability.

Seventh, disability was categorized by considering highest responses on the Likert-scale and did not consider potential combinations of disability. This was complemented by reporting scores within each category instead of reporting cut-offs defining disability categories. This approach provides one way for reporting disability without categorizing disability into a binary outcome. Eighth, due to time and resource constraints, collecting information on the experiences of caregivers and children with disabilities was not possible. This is an important area of future work to understand local context to generate data that can help monitor interventions and SDGs to include individuals with disabilities within education and work opportunities.

## Conclusion

This study provides an insight into factors associated with disability in children 5–17 years of age living at IM-HDSS and uses CFM as a standardized tool for assessment within communities. CFM helps to identify children who have varying level of disability. It allows assessment of disability in 13 domains, providing in-depth data on children. There is need to develop mechanisms for clinical assessment of children, especially those in the mild disability category, to allow for timely interventions. There is a possibility that these children are able to overcome their disability, which otherwise may progress further, thus limiting their level of integration in society. This study concludes that the lack of complete immunization and school enrollment are statistically and significantly associated with disability among children. This does not imply causal relation due to cross-sectional study design and requires further studies to get a better understanding of social issues related to access to education and health services by children with disabilities. Uganda has laws and policies which focus on individuals with disabilities; however, there is lack of their implementation. One of the potential reasons could be the lack of quality data. Implementation of CFM at IM-HDSS shows that it can be implemented for data collection at the sub-national level and can generate data that can be used by local district health offices to plan interventions catering to needs of children with disabilities, especially for education, employment and healthcare needs. These efforts can help in integration of individuals with disabilities and reduces their vulnerabilities. In addition to guiding development of interventions, data from CFM can be used for monitoring and evaluation of interventions, thus allowing policymakers and local district health officials to assess value for money associated with these interventions.

## Supporting information

S1 Table Factors associated with disability in children living at the IM-HDSS, Uganda (Logistic regression). (DOCX)
