# Supplementary material for: Understanding child disability: Factors associated with child disability at the Iganga-Mayuge Health and Demographic Surveillance Site in Uganda
Source: PLoS One. 2022 Apr 15;17(4):e0267182. doi: 10.1371/journal.pone.0267182 (PMC9012358; doi:10.1371/journal.pone.0267182)
Supplement: S1 Table — (DOCX) [file pone.0267182.s001.docx]

Supplementary table 1: Factors associated with disability in children living at the IM-HDSS, Uganda (Logistic Regression)

| **Characteristics** | **Mild versus combined moderate and severe** | | **Combined mild and moderate versus severe** | |
| --- | --- | --- | --- | --- |
|  | **Odd ratios (95% CI)** | **Coefficient (95% CI)** | **Odd ratios (95% CI)** | **Coefficient (95% CI)** |
| **Age groups** |  |  |  |  |
| 5-10 years | Reference | | Reference | |
| 11-14 years | **1.57*** (1.10 – 2.23) | 0.45 (0.09 – 0.80) | 1.56 (0.76 – 3.19) | 0.44 (-0.28 – 1.16) |
| 15-17 years | 1.38 (0.93 – 2.06) | 0.32 (-0.07 – 0.72) | 0.54 (0.23 – 1.30) | -0.61 (-1.48 – 0.26) |
| **Sex** |  |  |  |  |
| Male | Reference | | Reference | |
| Female | 0.89 (0.66 – 1.22) | -0.11 (-0.42 – 0.20) | 0.63 (0.33 – 1.20) | -0.47 (-1.12 – 0.18) |
| **Immunization status** |  |  |  |  |
| Completed | Reference | | Reference | |
| Not completed | **0.59*** (0.43 – 0.81) | **-0.53*** (-0.85 - -0.21) | **2.17*** (1.06 – 4.47) | **0.78*** (0.05 – 1.50) |
| **Currently in school** |  |  |  |  |
| Yes | Reference | | Reference | |
| No | **1.75*** (1.11 – 2.76) | **0.56*** (0.11 – 1.02) | **9.70*** (4.70 – 20.04) | **2.27*** (1.55 – 2.99) |
| **Have a primary caregiver** |  |  |  |  |
| Yes | Reference | | Reference | |
| No | **0.40*** (0.20 – 0.78) | **-0.92*** (-1.59 - -0.24) | 0.44 (0.10 – 1.92) | -0.83 (-2.31 – 0.65) |
| **Mother’s age at birth** |  |  |  |  |
| 14 – 20 years | Reference | | Reference | |
| 21 – 30 years | 0.90 (0.62 – 1.29) | -0.11 (-0.47 – 0.25) | 0.78 (0.36 – 1.67) | -0.25 (-1.01 – 0.52) |
| 31 – 40 years | 1.05 (0.66 – 1.67) | 0.05 (-0.41 – 0.51) | 1.18 (0.51 – 2.75) | 0.17 (-0.68 – 1.01) |
| 41 – 50 years | 1.21 (0.50 – 2.93) | 0.19 (-0.69 – 1.07) | 0.78 (0.14 – 4.30) | -0.25 (-1.96 – 1.46) |
| **Family system** |  |  |  |  |
| Single parent | Reference | | Reference | |
| Nuclear | 1.08 (0.60 – 1.93) | 0.07 (-0.51 – 0.66) | 0.60 (0.21 – 1.73) | -0.52 (-1.58 – 0.55) |
| Joint | 1.45 (0.78 – 2.68) | 0.37 (-0.25 – 0.99) | 0.51 (0.16 – 1.64) | - 0.66 (-1.82 – 0.49) |
| **Family size** | 1.01 (0.96 – 1.06) | 0.01 (-0.04 – 0.06) | 1.06 (0.96 – 1.16) | 0.06 (-0.038 – 0.15) |
| **Household wealth quintile** |  |  |  |  |
| Poorest | Reference | | Reference | |
| Poorer | 1.22 (0.81 – 1.86) | 0.20 (-0.21 – 0.62) | 0.92 (0.37 – 2.24) | -0.09 (-0.99 – 0.81) |
| Poor | 0.99 (0.65 – 1.52) | -0.01 (-0.43 – 0.42) | 1.35 (0.57 – 3.21) | 0.30 (-0.56 – 1.17) |
| Less poor | 0.89 (0.55 – 1.46) | -0.11 (- 0.60 – 0.38) | 1.25 (0.45 – 3.44) | 0.22 (-0.79 – 1.24) |
| Least poor | 0.86 (0.48 – 1.53) | -0.15 (-0.73 – 0.43) | 1.35 (0.44 – 4.16) | 0.30 (-0.82 – 1.43) |

*Statistically significant at p-value <0.05
